# Dietary patterns and indicators of cardiometabolic risk among rural adolescents: A cross-sectional study at 15-year follow-up of the MINIMat cohort

**DOI:** 10.3389/fnut.2023.1058965

**Published:** 2023-01-25

**Authors:** Mohammad Redwanul Islam, Syed Moshfiqur Rahman, Katarina Selling, Pieta Näsänen-Gilmore, Maria Kippler, Eero Kajantie, Anisur Rahman, Jesmin Pervin, Eva-Charlotte Ekström

**Affiliations:** ^1^Department of Women’s and Children’s Health, Uppsala University, Uppsala, Sweden; ^2^Maternal and Child Health Division, International Centre for Diarrhoeal Disease Research, Bangladesh (icddr,b), Dhaka, Bangladesh; ^3^Department of Public Health and Welfare, Finnish Institute for Health and Welfare, Helsinki, Finland; ^4^PEDEGO Research Unit, MRC Oulu, Oulu University Hospital, University of Oulu, Oulu, Finland; ^5^Institute of Environmental Medicine, Karolinska Institutet, Stockholm, Sweden

**Keywords:** dietary patterns, cardiometabolic risk markers, blood pressure, lipid profile, waist circumference, rural adolescents, low- and middle-income country (LMIC), Bangladesh

## Abstract

**Background:**

Diet being a modifiable factor, its relationship with cardiometabolic risk is of public health interest. The vast majority of studies on associations of dietary patterns with cardiometabolic risk indicators among adolescents are from high-income countries and urban settings. We sought to describe dietary patterns and examine their associations with selected cardiometabolic risk indicators–waist circumference (WC), systolic blood pressure, fasting lipid profile and insulin resistance–along with its gender stratification among adolescents in a low-income, rural setting.

**Methods:**

This cross-sectional study utilized data from the 15-year follow-up of the Maternal and Infant Nutrition Interventions in Matlab (MINIMat) cohort in southeast Bangladesh. The children who were born as singletons to the mothers randomized in the MINIMat trial and had valid birth anthropometrics were eligible for the follow-up. We employed a single, qualitative 24-hour recall to assess diet. Dietary patterns were derived from simple *K*-means cluster analysis, and calculation of dietary diversity score (DDS) using a validated instrument. Anthropometric parameters and systolic blood pressure were recorded. Fasting plasma triglyceride, total cholesterol, low- and high-density lipoproteins, insulin and glucose levels were measured. We calculated insulin resistance using the Homeostasis Model Assessment equation (HOMA-IR). Three right-skewed outcome variables were natural log (Ln) transformed: WC, triglyceride and HOMA-IR. Omnibus and gender-specific multiple linear regression models were fitted.

**Results:**

Among 2,253 adolescents (52.1% girls, 7.1% overweight/obese), we identified four diet clusters: Traditional, Fish-dominant, Meat-dominant, and High-variety. No significant associations were found between the clusters and indicators. On gender-stratification, triglyceride levels were lower among boys in the Fish-dominant (Ln-triglyceride β_adjusted_: −0.09; 95% confidence interval (CI): −0.15, −0.02) and Meat-dominant (Ln-triglyceride β_adjusted_: −0.08; 95% CI: −0.15, −0.004) clusters than among boys in the Traditional cluster. Compared to boys in the bottom quartile of DDS, boys in the top quartile had 2.1 mm of Hg (95% CI: 0.5, 3.6) higher systolic blood pressure and 1.9% (95% CI: 0.01–3.8%) higher WC.

**Conclusion:**

While statistically significant, the gender-specific differences in triglyceride, systolic blood pressure, and waist circumference across dietary patterns were small. Associations between dietary patterns and cardiometabolic risk indicators may require a time lag beyond mid-adolescence to manifest in a rural setting. Prospective studies are warranted to delineate the magnitude and direction of those associations.

## Introduction

Elevated systolic blood pressure (SBP) and raised blood levels of glucose and lipids pose a major challenge to global health. The Global Burden of Disease (GBD) 2019 study (1) examined the morbidity and mortality attributable to these cardiometabolic risks. SBP > 115 mm of Hg accounted for an estimated 10.8 million deaths worldwide in 2019 (1). The corresponding estimates for fasting plasma glucose >5.4 mmol/L and low-density lipoprotein (LDL) >1.3 mmol/L were 6.2 million and 2.4 million deaths, respectively (1). Over the past decade, the population exposure to cardiometabolic risks has increased sharply in low- and middle-income countries (LMICs). For instance, between 2010 and 2019, the age-standardized, risk-weighted prevalence of high fasting plasma glucose increased by about 2.5% annually in lower-middle income countries. In the same period, the annual rate of increase was 1.3% globally and 1.8% in high-income countries (1). The escalating exposure in LMICs coincides with rapid nutrition transition and changes in population diets due to widening availability of unhealthy foods (2). Diet is a modifiable factor intricately related to these cardiometabolic risks (3). Hence, exploring dietary patterns (DPs) in relation to indicators of cardiometabolic risk is relevant to understanding how habitual diet shapes cardiometabolic risk profile in different populations.

Cardiometabolic risk profile emerging in adolescence may track into adulthood. Epidemiological studies have shown that adverse cardiometabolic risk profile in adolescence predicts hypertension, type 2 diabetes, dyslipidemias and cardiovascular events decades later in the adulthood (4–6). Similarly, food choices and DPs established in adolescence have been found to persist well into adulthood (7, 8). Therefore, identification of healthful or deleterious DPs in terms of cardiometabolic risk among adolescents could inform non-communicable disease prevention strategies. Nevertheless, the vast majority of the studies investigating associations between DPs and indicators of cardiometabolic risk among adolescents are from high-income countries (9–14) or urban settings of middle-income countries (15–19). These studies are unlikely to have captured the potentially higher biological susceptibility (20) from early-life undernutrition (21, 22) among rural adolescents of LMICs. Furthermore, the findings are heterogeneous, particularly regarding gender-based differences in association. At 14-year follow-up of an Australian birth cohort, a DP with higher shares of saturated fat, refined sugar and added salt was cross-sectionally associated with higher total cholesterol (TC) and waist circumference (WC) among girls only (9). Prospective analyses in the same cohort revealed positive associations of an “energy dense, high fat, and low fiber” DP with WC among girls and fasting glucose among boys at 17 years (11). In a cross-sectional study among Tunisian adolescents (*n* = 1,019, 44.4% rural), a “modern” DP consisting of white bread, high-fat dairy, sugar-sweetened beverage (SSB) and processed meat was positively associated with WC only among the boys (23). These diverse findings indicate the need for studying DPs in relation to cardiometabolic risk among adolescents in resource-limited, rural settings for context-specific empirical evidence.

Bangladesh is a lower-middle-income country in South Asia and home to 36 million adolescents, who represent one-fifth of the population. The majority of these adolescents live in rural areas (24). Yet studies on DPs and cardiometabolic risk among rural adolescents in Bangladesh remain markedly sparse. Aiming to address this gap, we sought to: (i) describe DPs in a rural birth cohort of Bangladeshi adolescents; and (ii) examine the associations of the DPs with WC, SBP, plasma levels of triglyceride (TG), TC, LDL and high-density lipoprotein (HDL), and insulin resistance (IR) along with potential difference in associations by gender.

## Materials and methods

### Study design, participants, and setting

**FIGURE 1:**
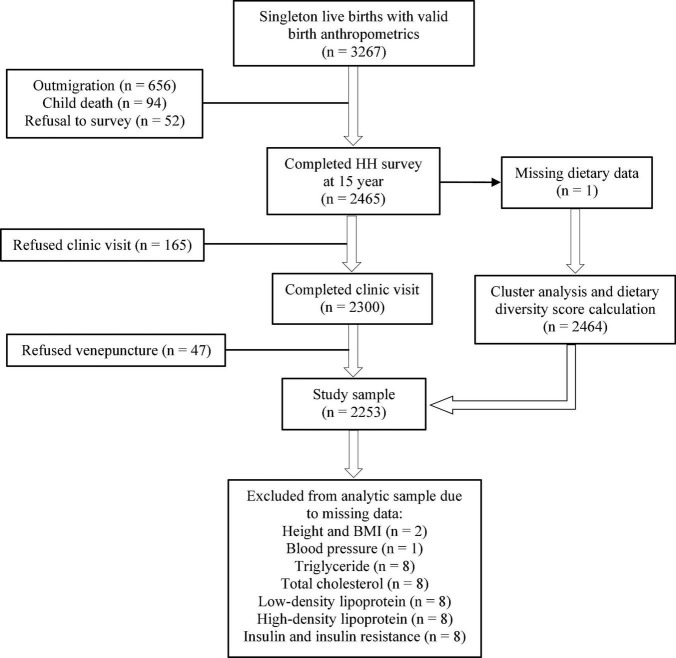
Flowchart for inclusion of Maternal and Infant Nutrition Intervention in Matlab (MINIMat) adolescents into the present study. HH, household.

This cross-sectional study availed data collected during the 15-year follow-up of MINIMat trial, conducted from September 2017 to June 2019. MINIMat (Maternal and Infant Nutrition Interventions in Matlab, reg#ISRCTN16581394) was a community-based, randomized trial that tested the effects of prenatal food and micronutrient supplementation on maternal and birth outcomes (25). Between 2001 and 2003, a total of 4,436 pregnant women from Matlab were randomized. This resulted in 3,267 singleton live births with valid birth anthropometrics, forming the MINIMat cohort that has been intensively followed up (26). The 15-year follow-up comprised three parts: formative phase, household survey, and clinic visit. Trained interviewers with at least 12 years of formal education interviewed the adolescent-mother/guardian dyads at their houses using a pre-tested, structured questionnaire. The clinic visit involved anthropometric assessment and collection of fasting blood sample. Out of the 3,267 eligible adolescents, 2,465 (75.5%) completed the household survey and 2,300 (70.4%) completed the clinic visit. The participant flow and reasons for loss to follow-up are presented in Figure 1, “Results” section.

Matlab is a rural sub-district, located about 55 km to the southeast of the capital city of Dhaka. The community is agrarian and rice farming is the main occupation in Matlab, but a few villages rely on fishing as the means of income. An agricultural lean period usually occurs from September to November (27).

### Dietary assessment

The foods consumed by Matlab adolescents were listed during the formative phase. Focus groups (28) and findings from previous follow-ups informed the listing process. There were 15 food groups in total, and the items included in each are presented in Supplementary Table 1. The details of the food grouping have been reported elsewhere (29, 30). Briefly, the foods other than ultra-processed and deep-fried foods were arranged into 10 groups using locally adapted version of a validated (31, 32) instrument endorsed by the Food and Agriculture Organization (33). The groups were: (i) grains, white roots and tubers, and plantains; (ii) vitamin A-rich vegetables, tubers and fruits; (iii) dark green leafy vegetables; (iv) other vegetables; (v) other fruits; (vi) meat; (vii) egg; (viii) fish; (ix) legumes, nuts and seeds; and (x) milk products. The ultra-processed foods (UPFs) were identified using the NOVA system proposed by Monteiro et al. (34), and consolidated into four groups: (i) ready-to-eat or “instant” foods; (ii) confectionery, sweets and similar packaged products; (iii) savory snacks, and (iv) sugar-sweetened beverage (SSB) including energy drinks. Foods submerged in heated oil while cooking were grouped as deep-fried foods.

A single, interactive, 24-hour, qualitative recall was employed to assess consumption. Reported consumption of one tablespoonful (∼15 grams) or more of ≥1 item(s) belonging to a food group classified the adolescent as a consumer (coded “1”). Those who did not consume received a code of “0” for that food group. The one-tablespoon requirement was used to avoid trivial consumption from inflating the heterogeneity in DPs (35). The enumerators probed for unreported consumption after the initial recall using list and pictorial aid displaying charted photographs of foods. This double-pass approach has been recommended to minimize recall bias (36).

### Assessment of cardiometabolic risk indicators

Trained nurses collected data following a standardized protocol. Body weight was recorded with a digital scale (Tanita BC-418 Body Composition Analyzer, 0.2 kg) and height with a stadiometer (Seca 214, 0.1 cm) while adolescents wore standard light clothes provided by the project and were barefoot. The weight of the clothes (200 gram) were deducted from the measured weight. Body mass index (BMI) was calculated by dividing body weight with height squared (kg/m^2^). BMI-for-age z-score (BAZ) was calculated using the World Health Organization reference (37). For descriptive purpose, we categorized the adolescents as thin (BAZ < −2), normal-weight (−2 ≤ BAZ ≥ + 1), and overweight/obese (BAZ > + 1). WC was measured midway between the lower margin of the least palpable rib and iliac crest with a non-elastic tape (TALC) to the nearest 0.1 cm. Sitting BP was measured in triplicates, at two-minute intervals, using an Omron M10 device after a 10-min seated rest. Venous blood samples (6 ml) after an overnight fast were collected in Lithium-heparin tubes (Sarstedt). The samples were centrifuged; plasma was separated, aliquoted and stored at −70°C. Plasma TC, TG, LDL, and HDL levels were measured in a Cobas Analyzer (Roche) through enzymatic colorimetric assay at the laboratory of the Department of Clinical Chemistry, Skåne University Hospital, Sweden. Plasma insulin level was measured at the icddr,b laboratory. Fasting glucose was measured using the Contour TS Blood Glucose Monitoring System (Bayer) during the clinic visit. We calculated IR using the Homeostasis Model Assessment (HOMA) equation: HOMA-IR = (fasting insulin mU/L × fasting glucose in mmol/L) ÷ 22.5 (38).

### Socio-demographic variables

Gender was a dichotomous variable (girl/boy). An asset score was calculated for each household from principal component analysis (39) of the data on ownership of a set of durables (e.g., mobile phone, television, refrigerator, etc.), access to electricity and sanitary latrine, and nature of fuel used. We constructed a categorical variable (household wealth) by converting asset scores into tertiles: the lowest, intermediate and highest tertiles representing the poorest, middle-status and richest households, respectively. Educational status was categorized according to completed years of formal education: none, primary (1–5 years), and secondary (6–12 years) for adolescents or secondary and above (≥6 years) for mothers.

### Statistical analysis

We adopted a predefined, *a priori* approach and a data-driven, *a posteriori* approach (40, 41) to derive DPs. The *a priori* approach entailed calculation of individual dietary diversity score (DDS) (33) by summing up the codes received for the 10 food groups (excluding five groups of ultra-processed and deep-fried foods). DDS could range from 0–10. A categorical variable representing quartiles of DDS was created. The *a posteriori* approach involved cluster analysis (40, 41), given our interest in data-driven grouping of the adolescents into mutually exclusive clusters based on consumption. Cluster analysis assigns participants with similar consumption pattern to the same cluster using Euclidean distance metrics (42). We implemented Simple *K*-means Clustering algorithm in Weka (maximum 500 iterations) (43) with 14 food groups. The “grains, white roots and tubers, and plantains” group was excluded as 99% of the adolescents consumed rice from that group, making it the least discriminative dietary variable. The algorithm generated cluster centroids representing proportions of consuming adolescents by food groups in that cluster. A line graph was created by plotting the cluster sums of squared errors against cluster numbers ranging from 2–10 (Supplementary Figure 1) to help decide the optimal number of clusters. The point where the slope of the line levels off (the “elbow”) indicates the optimal number of clusters. We determined the number of clusters indicating DPs to be four based on the plot, cluster size and interpretability in Matlab context. The clusters were given descriptive labels and a categorical variable was created for further analysis.

Categorical variables are described with frequency and percentage, and continuous variables with mean and standard deviation (SD) or median and interquartile range. We checked the distributions of the continuous variables by examining histograms and quantile-quantile plots. The following right-skewed variables were natural log (Ln) transformed: WC, TG, and HOMA-IR. Means across the clusters and DDS quartiles were compared with one-way analysis of variance (ANOVA). Linear regression models–for all participants and gender-specific–were fitted, and regression coefficients with 95% confidence intervals (CI) are reported. We examined quantile-quantile plots of the residuals and residuals versus fitted plots to rule out violation of assumptions. The adjusted models accounted for gender, household wealth and maternal education based on a directed acyclic graph (Supplementary Figure 2). We did not adjust for BMI considering it a mediator in the DP-cardiometabolic indicator relationship. All tests were two-tailed and *P*-values < 0.05 were considered statistically significant. The analyses were performed in R, version 4.1.2 (44). In this study, a sample size of 2,253 provides 80% power (two-tailed with alpha = 0.05) to detect a difference of 0.06 SD.

### Ethics approval

The 15-year follow-up has been approved by the Ethical Review Committee at icddr,b in Dhaka, Bangladesh (PR–17029; date 2017/05/23). An additional approval has been obtained from the Ethics Review Authority (*Etikprövningsmyndigheten*) in Sweden (2021–02796; date 2021/11/15). We obtained written informed consent from the mothers and assent from the adolescents. The study was carried out in accordance with the Declaration of Helsinki.

## Results

Of the 3,267 eligible adolescents, 2,465 completed the household survey. Loss to follow-up resulted from: outmigration (*n* = 656), child death (*n* = 94), and refusal to household visit (*n* = 52). Excluding one participant with missing diet data, the cluster analysis and calculation of DDS involved 2,464 adolescents. The clinic visit was refused by 165 adolescents and another 47 refused venepuncture. Thus, the study sample comprised 2,253 adolescents. Adolescents with missing data on height, and hence, BMI (*n* = 1); blood pressure (*n* = 1); TG (*n* = 8); TC (*n* = 8); LDL (*n* = 8); HDL (*n* = 8); and insulin, and hence, HOMA-IR (*n* = 8) were excluded from analyses involving the respective variables (Figure 1).

### Description of the diet clusters

The four clusters were labeled as follows: Traditional (*n* = 832, 33.8%), Fish-dominant (*n* = 604, 24.5%), Meat-dominant (*n* = 514, 20.9%), and High-variety (*n* = 514, 20.9%). The cluster centroids for each food group–which represent the proportions of adolescents who consumed item(s) from that group–are presented in Table 1.

**TABLE 1:** Proportion of adolescents who consumed at least one tablespoonful (∼15 grams) of ≥1 item(s) from the 14 food groups by diet clusters.

Food group	All (*n* = 2,464)	Traditional cluster (*n* = 832)	Fish-dominant cluster (*n* = 604)	Meat-dominant (*n* = 514)	High-variety cluster (*n* = 514)
Vitamin A-rich^1^ fruits, vegetables, and tubers	0.314	0.252	0.214	0.241	0.605
Dark green leafy vegetables	0.268	0.279	0.252	0.214	0.323
Other (non-vitamin-A-rich) vegetables	0.607	0.739	0.712	0.148	0.731
Other (non-vitamin-A-rich) fruits	0.452	0.296	0.364	0.381	0.877
Flesh and organ meat	0.352	0.139	0.238	0.765	0.418
Egg	0.349	0.279	0.316	0.333	0.517
Fish	0.732	0.907	0.924	0.179	0.774
Nuts, seeds, and legumes	0.462	0.346	0.290	0.465	0.850
Dairy	0.305	0.239	0.237	0.249	0.548
Ready-to-eat and “instant” foods (UPF)	0.130	0.112	0.134	0.095	0.189
Confectionery, sweets, and similar packaged products (UPF)	0.534	0.475	0.498	0.451	0.757
Savory snacks (UPF)	0.354	0.266	0.379	0.292	0.529
Sugar-sweetened beverage (UPF)	0.120	0.055	0.111	0.103	0.251
Deep-fried foods	0.413	0	1	0.278	0.529

UPF, ultra-processed foods.

^1^Based on the FAO guideline (35) that defines Vitamin A-rich fruits, vegetables, and tubers as those containing at least 120 Retinol Equivalent per 100 gram.

The Traditional cluster was characterized by a high reported consumption of fish along with low-carotenoid vegetables. About 47.5% of those in this cluster consumed packaged confectionery. The proportions of adolescents consuming animal-source foods (excluding fish) and dark green and vitamin-A-rich vegetables and fruits were fairly low in the cluster. Considering the pattern in Traditional cluster reflective of rural Bangladeshi diets, it was chosen as the reference category in further analyses. The proportion of adolescents consuming fish was highest in the Fish-dominant cluster (92.4%). Consumption of deep-fried foods was universal in this cluster and nearly 50% of the adolescents also consumed packaged confectionery. The Meat-dominant cluster was distinguished by the highest proportion of adolescents consuming meat (76.5%). Consumption of legumes (46.5%) and packaged confectionery (45.1%) was high as well, whereas that of fish was markedly low (17.9%). The proportion of adolescents with consumption exceeded 50% for 10 out of the 14 food groups in the High-variety cluster. Three-fourths of the adolescents in this cluster consumed packaged confectionery and about 53% consumed savory snacks and deep-fried foods. Consumption of low-carotenoid fruits, nuts, seeds, and legumes was also high.

The mean DDS across the clusters were as follows: Traditional (4.4; 95% CI: 4.3–4.5), Fish-dominant (4.5; 95% CI: 4.4–4.6), Meat-dominant (3.9; 95% CI: 3.8–4.0), and High-variety (6.6; 95% CI: 6.5–6.7). The mean BAZ of the adolescents in these clusters were as follows: Traditional (−0.89; 95% CI: −0.98, −0.80), Fish-dominant (−0.91; 95% CI: −1.01, −0.81), Meat-dominant (−1.01; 95% CI: −1.12, −0.89), and High-variety (−0.96; 95% CI: −1.08, −0.84).

### Characteristics of the study participants

Table 2 demonstrates the socio-demographic and anthropometric characteristics and cardiometabolic profile of the MINIMat adolescents. The girls were slightly over-represented in the sample (52.1%). On average, the boys were taller and heavier than the girls; but the median BMI was higher among the girls. Overweight/obesity was more prevalent among the girls than the boys (7.9 versus 6.2%). Approximately 40% of the girls belonged to the poorest households, whereas 28.5% of the boys came from the poorest households. There were more girls than boys in the Traditional cluster and in the first quartile of DDS. The median WC in the sample was 62.4 cm and it did not differ by gender.

**TABLE 2:** Descriptive characteristics of the adolescents participating in the study.

Characteristics	All (*n* = 2253)		Boys (*n* = 1079)		Girls (*n* = 1174)		*P* ^1^
	*n*	Value	*n*	Value	*n*	Value	
Age (years)	2,253	15.0 (0.1)	1,079	15.0 (0.1)	1,174	15.0 (0.1)	0.382
Height (cm)	2,251	156.5 (7.6)	1,078	160.5 (7.7)	1,173	152.9 (5.4)	**<0.001**
Weight (kg)	2,253	43.9 (39.3–49.7)	1,079	45.3 (39.3–51.0)	1,174	43.1 (39.2–48.5)	**<0.001**
BMI (kg/m^2^)	2,251	17.8 (16.3–19.8)	1,078	17.2 (15.9–18.9)	1,173	18.4 (16.9–20.5)	**<0.001**
BAZ categories^2^	2,251						**<0.001**
Normal	1,649	73.3	707	65.6	942	80.3	
Thin	442	19.6	304	28.2	138	11.8	
Overweight/obese	160	7.1	67	6.2	93	7.9	
Household wealth	2,253						**<0.001**
Poorest	775	34.4	308	28.5	467	39.8	
Intermediate	723	32.1	397	36.8	326	27.8	
Richest	755	33.5	374	34.7	381	32.4	
Maternal education	2,253						**0.027**
None	447	19.8	210	19.5	237	20.2	
Primary	800	35.5	357	33.1	443	37.7	
Secondary or above	1,006	44.7	512	47.4	494	42.1	
Adolescent education							**<0.001**
None	355	15.8	214	19.8	141	12.0	
Primary	57	2.5	45	4.2	12	1.0	
Secondary	1,841	81.7	820	76.0	1,021	87.0	
Diet clusters	2,253						**<0.001**
Traditional	764	33.9	282	26.1	482	41.0	
Fish-dominant	559	24.8	316	29.3	243	20.7	
Meat-dominant	468	20.8	222	20.6	246	21.0	
High-variety	462	20.5	259	24.0	203	17.3	
DDS quartiles	2,253						**0.026**
Q1 (DDS ≤ 4)	965	42.8	439	40.7	526	44.8	
Q2 (DDS = 5)	568	25.2	262	24.3	306	26.1	
Q3 (DDS = 6)	427	19.0	227	21.0	200	17.0	
Q4 (DDS ≥ 7)	293	13.0	151	14.0	142	12.1	
DDS	2,253	4.8 (1.5)	1,079	4.9 (1.6)	1,174	4.7 (1.4)	
WC (cm)	2,253	62.4 (58.9–66.8)	1,079	62.4 (59.3–66.4)	1,174	62.4 (58.8–67.2)	0.989
SBP (mm of Hg)	2,252	108.8 (8.0)	1,078	110.3 (8.2)	1,174	107.4 (7.6)	**<0.001**
TG (mmol/L)	2,245	0.9 (0.7–1.2)	1,077	0.9 (0.7–1.1)	1,168	1.0 (0.8–1.3)	**<0.001**
TC (mmol/L)	2,245	3.6 (0.7)	1,077	3.4 (0.6)	1,168	3.7 (0.7)	**<0.001**
LDL (mmol/L)	2,245	2.1 (0.6)	1,077	2.0 (0.5)	1,168	2.2 (0.6)	**<0.001**
HDL (mmol/L)	2,245	1.0 (0.2)	1,077	1.0 (0.2)	1,168	1.0 (0.2)	0.441
Insulin (μU/L)	2,245	11.4 (7.8–18.3)	1,078	9.6 (6.6–16.3)	1,167	12.9 (9.3–20.2)	**<0.001**
Glucose (mmol/L)	2,253	5.2 (0.5)	1,079	5.3 (0.5)	1,174	5.1 (0.4)	**<0.001**
HOMA-IR	2,245	2.6 (1.7–4.2)	1,078	2.2 (1.5–3.8)	1,167	2.9 (2.1–4.7)	**<0.001**

BMI, body mass index; BAZ, BMI-for-age z-score; WC, waist circumference; DDS, dietary diversity score; Q, quartile; SBP, systolic blood pressure; TG, triglyceride; TC, total cholesterol; LDL, low-density lipoprotein; HDL, high-density lipoprotein; HOMA-IR, insulin resistance from Homeostasis Model Assessment.

Values represent percentage for categorical variables, mean with standard deviation for continuous variables (approximately) normally distributed, or median with inter-quartile range for continuous variables that were skewed. *P*-values in bold indicate statistical significance.

Missing data: height and BMI (*n* = 2), SBP (*n* = 1), TG (*n* = 8), TC (*n* = 8), LDL (*n* = 8), HDL (*n* = 8), and insulin and HOMA-IR (*n* = 8).

^1^P-value for gender difference from Chi-squared test, independent samples *t*-test, or Wilcoxon rank-sum test.

^2^BAZ below −2 SD and above + 1 SD define thinness and overweight/obesity, respectively.

### Bivariate analysis

The mean levels of WC, SBP, and blood markers by cluster and DDS quartile are presented in Table 3. There was a small but statistically significant difference in TG level by cluster. Adolescents in the Traditional cluster had the highest mean TG (geometric mean 0.99 mmol/L, 95% CI: 0.96–1.02), while those in the Fish-dominant cluster had the lowest mean TG (geometric mean 0.92 mmol/L, 95% CI: 0.89–0.95). Small but statistically significant differences in means of WC, SBP, and HDL were observed across DDS quartiles.

**TABLE 3:** Levels of cardiometabolic risk markers by diet clusters and quartiles of dietary diversity score.

Diet clusters	WC^1^ (cm)	SBP (mm of Hg)	TG^1^ (mmol/L)	TC (mmol/L)	LDL (mmol/L)	HDL (mmol/L)	HOMA-IR^1^
**Mean (95% confidence interval)**							
Traditional	63.3 (62.8–63.8)	108.5 (107.9–109.0)	0.99 (0.96–1.02)	3.62 (3.57–3.67)	2.16 (2.12–2.20)	1.02 (1.004–1.04)	3.1 (2.9–3.2)
Fish-dominant	63.6 (63.0–64.2)	109.0 (108.3–109.6)	0.92 (0.89–0.95)	3.54 (3.49–3.59)	2.12 (2.07–2.16)	1.02 (1.004–1.04)	2.8 (2.6–2.9)
Meat-dominant	63.0 (62.4–63.6)	108.4 (107.7–109.2)	0.94 (0.91–0.98)	3.54 (3.48–3.60)	2.11 (2.06–2.16)	1.01 (0.99–1.03)	2.8 (2.6–3.0)
High-variety	63.5 (62.9–64.1)	109.5 (108.8–110.3)	0.93 (0.89–0.96)	3.55 (3.48–3.61)	2.12 (2.07–2.18)	1.01 (0.99–1.03)	3.0 (2.8–3.2)
*P* ^2^	0.524	0.115	**0.003**	0.086	0.424	0.779	0.070
**DDS quartiles**							
Q1 (DDS ≤ 4)	63.2 (62.7–63.6)	108.2 (107.7–108.8)	0.96 (0.93–0.98)	3.57 (3.53–3.61)	2.13 (2.09–2.17)	1.01 (1.001–1.03)	2.8 (2.7–3.0)
Q2 (DDS = 5)	63.5 (63.0–64.1)	109.2 (108.6–109.9)	0.97 (0.94–1.00)	3.57 (3.52–3.62)	2.13 (2.08–2.17)	1.01 (0.99–1.03)	3.1 (2.9–3.3)
Q3 (DDS = 6)	62.9 (62.3–63.5)	108.7 (108.0–109.4)	0.92 (0.89–0.96)	3.57 (3.50–3.64)	2.12 (2.06–2.18)	1.04 (1.02–1.07)	2.9 (2.7–3.1)
Q4 (DDS ≥ 7)	64.3 (63.5–65.1)	110.0 (109.1–111.0)	0.94 (0.89–0.98)	3.56 (3.48–3.64)	2.14 (2.07–2.21)	0.99 (0.97–1.02)	3.0 (2.7–3.2)
*P* ^2^	**0.03**	**0.005**	0.275	0.992	0.962	**0.017**	0.076

WC, waist circumference; SBP, systolic blood pressure; TG, triglyceride; TC, total cholesterol; LDL, low-density lipoprotein; HDL, high-density lipoprotein; HOMA-IR, insulin resistance from Homeostasis Model Assessment; DDS, dietary diversity score; Q, quartile. *P*-values in bold indicate statistical significance.

^1^WC, TG, and HOMA-IR were right skewed and (natural) log transformed, and their geometric means with 95% confidence intervals reported here.

^2^From one-way analysis of variance.

### Associations of diet clusters with cardiometabolic risk indicators

Table 4 shows the regression coefficients with 95% CIs from crude and adjusted linear models for associations between the clusters and cardiometabolic risk indicators. Overall, the diet clusters were not associated with the indicators. On gender stratification, TG levels were significantly lower among boys in the Fish-dominant cluster (Ln TG β_*adj*_: −0.089; 95% CI: −0.155, −0.022) and Meat-dominant cluster (Ln TG β_*adj*_: −0.076; 95% CI: −0.149, −0.004) compared to boys in the Traditional cluster. No statistically significant association was observed among the girls.

**TABLE 4:** Overall and gender-stratified associations of diet clusters with anthropometric and blood markers of cardiometabolic risk among the Maternal and Infant Nutrition Intervention in Matlab (MINIMat) adolescents.

Marker	Diet clusters	All (*N* = 2,253)		Boys (*N* = 1,079)		Girls (*N* = 1,174)	
		β (95% CI)	*P*	β (95% CI)	*P*	β (95% CI)	*P*
Ln WC Crude model	Traditional	Ref		Ref		Ref	
	Fish-dominant	0.005 (−0.007, 0.016)	0.435	0.007 (−0.009, 0.024)	0.376	0.003 (−0.014, 0.019)	0.766
	Meat-dominant	−0.005 (−0.017, 0.007)	0.434	−0.003 (−0.021, 0.015)	0.720	−0.006 (−0.022, 0.011)	0.517
	High-variety	0.003 (−0.010, 0.015)	0.674	0.007 (−0.010, 0.025)	0.402	−0.002 (−0.020, 0.016)	0.835
Ln WC Adjusted^1^ model	Traditional	Ref		Ref		Ref	
	Fish-dominant	0.002 (−0.009, 0.013)	0.724	0.009 (−0.007, 0.025)	0.281	−0.003 (−0.019, 0.014)	0.739
	Meat-dominant	−0.007 (−0.019, 0.004)	0.221	−0.004 (−0.021, 0.014)	0.669	−0.009 (−0.025, 0.007)	0.284
	High-variety	−0.004 (−0.016, 0.009)	0.567	0.002 (−0.015, 0.019)	0.840	−0.008 (−0.026, 0.009)	0.356
SBP Crude model	Traditional	Ref		Ref		Ref	
	Fish-dominant	0.473 (−0.403, 1.348)	0.290	−0.007 (−1.332, 1.317)	0.992	−0.120 (−1.290, 1.049)	0.840
	Meat-dominant	−0.051 (−0.975, 0.873)	0.913	−0.221 (−1.674, 1.231)	0.765	−0.432 (−1.596, 0.733)	0.467
	High-variety	**1.049 (0.121, 1.976)**	**0.027**	0.815 (−0.577, 2.206)	0.251	0.171 (−1.072, 1.415)	0.787
SBP Adjusted^1^ model	Traditional	Ref		Ref		Ref	
	Fish-dominant	−0.130 (−1.002, 0.742)	0.770	0.047 (−1.275, 1.369)	0.944	−0.169 (−1.346, 1.008)	0.778
	Meat-dominant	−0.405 (−1.319, 0.508)	0.384	−0.263 (−1.713, 1.188)	0.722	−0.443 (−1.614, 0.728)	0.458
	High-variety	0.356 (−0.570, 1.281)	0.451	0.602 (−0.796, 2.001)	0.398	0.098 (−1.155, 1.350)	0.878
Ln TG Crude model	Traditional	Ref		Ref		Ref	
	Fish-dominant	−0.076 (−0.121, −0.032)	<0.001	**−0.089 (−0.155, −0.023)**	**0.008**	−0.007 (−0.069, 0.054)	0.814
	Meat-dominant	−0.049 (−0.096, −0.002)	0.041	**−0.076 (−0.148, −0.004)**	**0.039**	−0.003 (−0.063, 0.058)	0.932
	High-variety	−0.067 (−0.114, −0.020)	0.005	−0.052 (−0.121, 0.017)	0.140	−0.038 (−0.102, 0.027)	0.257
Ln TG Adjusted^1^ model	Traditional	Ref		Ref		Ref	
	Fish-dominant	−0.045 (−0.089, 0.00007)	0.050	**−0.089 (−0.155, −0.022)**	**0.009**	0.001 (−0.060, 0.062)	0.974
	Meat-dominant	−0.031 (−0.077, 0.016)	0.196	**−0.076 (−0.149, −0.004)**	**0.039**	0.006 (−0.055, 0.067)	0.846
	High-variety	−0.032 (−0.079, 0.015)	0.181	−0.053 (−0.122, 0.017)	0.140	−0.026 (−0.091, −0.032)	0.439
TC Crude model	Traditional	Ref		Ref		Ref	
	Fish-dominant	**−0.078 (−0.151, −0.005)**	**0.037**	−0.025 (−0.121, 0.071)	0.614	−0.005 (−0.113, 0.103)	0.924
	Meat-dominant	**−0.081 (−0.158, −0.003)**	**0.040**	−0.064 (−0.170, 0.041)	0.229	−0.034 (−0.142, 0.073)	0.527
	High-variety	−0.070 (−0.147, 0.007)	0.077	−0.026 (−0.127, 0.074)	0.606	0.009 (−0.105, 0.124)	0.875
TC Adjusted^1^ model	Traditional	Ref		Ref		Ref	
	Fish-dominant	−0.019 (−0.091, 0.053)	0.610	−0.024 (−0.120, 0.071)	0.615	−0.009 (−0.118, 0.010)	0.864
	Meat-dominant	−0.044 (−0.120, 0.031)	0.248	−0.060 (−0.165, 0.045)	0.262	−0.029 (−0.136, 0.079)	0.600
	High-variety	−0.013 (−0.089, 0.064)	0.746	−0.032 (−0.133, 0.070)	0.539	0.006 (−0.109, 0.121)	0.921
LDL Crude model	Traditional	Ref		Ref		Ref	
	Fish-dominant	−0.041 (−0.103, 0.022)	0.202	0.007 (−0.073, 0.088)	0.855	0.016 (−0.078, 0.110)	0.743
	Meat-dominant	−0.049 (−0.115, 0.017)	0.146	−0.025 (−0.113, 0.063)	0.579	−0.019 (−0.112, 0.074)	0.685
	High-variety	−0.035 (−0.101, 0.031)	0.296	0.008 (−0.076, 0.092)	0.850	0.022 (−0.077, 0.122)	0.657
LDL Adjusted^1^ model	Traditional	Ref		Ref		Ref	
	Fish-dominant	0.006 (−0.056, 0.067)	0.860	0.008 (−0.072, 0.087)	0.853	0.009 (−0.085, 0.103)	0.854
	Meat-dominant	−0.021 (−0.085, 0.044)	0.531	−0.021 (−0.109, 0.066)	0.631	−0.017 (−0.110, 0.077)	0.728
	High-variety	0.007 (−0.058, 0.072)	0.844	−0.003 (−0.087, 0.082)	0.952	0.016 (−0.084, 0.115)	0.759
HDL Crude model	Traditional	Ref		Ref		Ref	
	Fish-dominant	0.002 (−0.022, 0.026)	0.867	0.003 (−0.033, 0.039)	0.876	−0.005 (−0.040, 0.029)	0.761
	Meat-dominant	−0.011 (−0.036, 0.015)	0.402	−0.016 (−0.055, 0.023)	0.429	−0.009 (−0.043, 0.025)	0.593
	High-variety	−0.006 (−0.031, 0.020)	0.664	−0.023 (−0.061, 0.014)	0.228	0.010 (−0.026, 0.047)	0.573
HDL Adjusted^1^ model	Traditional	Ref		Ref		Ref	
	Fish-dominant	−0.00001 (−0.025, 0.025)	0.998	0.002 (−0.034, 0.038)	0.913	−0.007 (−0.042, 0.028)	0.691
	Meat-dominant	−0.011 (−0.036, 0.015)	0.418	−0.013 (−0.053, 0.026)	0.498	−0.010 (−0.044, 0.024)	0.558
	High-variety	−0.006 (−0.032, 0.020)	0.646	−0.016 (−0.054, 0.022)	0.408	0.008 (−0.029, 0.045)	0.673
Ln HOMA-IR Crude model	Traditional	Ref		Ref		Ref	
	Fish-dominant	**−0.103 (−0.188, −0.018)**	**0.017**	−0.092 (−0.222, 0.037)	0.163	−0.008 (−0.121, 0.104)	0.884
	Meat-dominant	−0.087 (−0.176, 0.002)	0.057	−0.072 (−0.214, 0.070)	0.322	−0.054 (−0.165, 0.057)	0.342
	High-variety	−0.033 (−0.122, 0.057)	0.476	0.019 (−0.117, 0.156)	0.781	0.003 (−0.116, 0.122)	0.958
Ln HOMA-IR Adjusted^2^ model	Traditional	Ref		Ref		Ref	
	Fish-dominant	−0.055 (−0.138, 0.027)	0.189	−0.090 (−0.218, 0.038)	0.170	−0.026 (−0.135, 0.082)	0.634
	Meat-dominant	−0.052 (−0.139, 0.034)	0.236	−0.068 (−0.209, 0.072)	0.340	−0.040 (−0.148, 0.068)	0.464
	High-variety	−0.028 (−0.116, 0.059)	0.527	−0.016 (−0.152, 0.120)	0.814	−0.043 (−0.158, 0.072)	0.464

Ref, reference category; Ln, natural-log transformed variable where the base of the log was 2.71828; b, regression coefficient; WC, waist circumference; SBP, systolic blood pressure; TG, triglyceride; TC, total cholesterol; LDL, low-density lipoprotein; HDL, high-density lipoprotein; HOMA-IR, insulin resistance from Homeostasis Model Assessment.

^1^Adjusted for gender, household wealth, and maternal education.

^2^Additionally adjusted for sample storage time, that showed a negative correlation with fasting insulin level (Spearman’s ρ = −0.2, *P* < 0.001). Statistically significant coefficients are presented in bold.

### Associations of DDS quartiles with cardiometabolic risk indicators

The adjusted linear regression models analyzing associations between DDS quartiles and cardiometabolic risk indicators are presented in Table 5. On average, those in the second (DDS = 5) and top (DDS ≥ 7) quartiles had 0.9 (95% CI: 0.1–1.7) and 1.4 (95% CI: 0.4–2.4) mm of Hg higher SBP, respectively, than their peers in the bottom quartile (DDS ≤ 4). Although on gender stratification the association disappeared among the girls, it persisted among the boys (β_*adj*_ for boys in the top quartile: 2.1; 95% CI: 0.5–3.6). Compared to the boys in the bottom quartile, WC was 1.9% (95% CI: 0.01–3.8%) higher among the boys in the top quartile. Boys in the third quartile (DDS = 6) had 0.04 mmol/L (95% CI: 0.01–0.08) higher HDL than the boys in the bottom quartile. These associations were not observed among the girls.

**TABLE 5:** Overall and gender-stratified associations of dietary diversity score (DDS) quartiles with anthropometric and blood markers of cardiometabolic risk among the Maternal and Infant Nutrition Intervention in Matlab (MINIMat) adolescents.

Marker		All (*N* = 2,253)		Boys (*N* = 1,079)		Girls (*N* = 1,174)	
		β (95% CI)	*P*	β (95% CI)	*P*	β (95% CI)	*P*
Ln WC Adjusted^1^ model	Q1 (DDS ≤ 4)	Ref		Ref		Ref	
	Q2 (DDS = 5)	0.003 (−0.008, 0.013)	0.604	0.012 (−0.003, 0.027)	0.133	−0.005 (−0.020, 0.010)	0.550
	Q3 (DDS = 6)	−0.009 (−0.020, 0.003)	0.153	−0.012 (−0.028, 0.004)	0.140	−0.005 (−0.022, 0.013)	0.598
	Q4 (DDS ≥ 7)	0.009 (−0.004, 0.023)	0.178	**0.019 (0.0001, 0.037)**	**0.048**	−0.001 (−0.020, 0.019)	0.945
SBP Adjusted^1^ model	Q1 (DDS ≤ 4)	Ref		Ref		Ref	
	Q2 (DDS = 5)	**0.913 (0.094, 1.732)**	**0.029**	1.158 (−0.010, 2.415)	0.071	0.706 (−0.367, 1.779)	0.197
	Q3 (DDS = 6)	0.140 (−0.764, 1.044)	0.761	0.167 (−1.157, 1.492)	0.804	0.092 (−1.148, 1.333)	0.884
	Q4 (DDS ≥ 7)	**1.413 (0.373, 2.453)**	**0.008**	**2.078 (0.543, 3.612)**	**0.008**	0.704 (−0.710, 2.118)	0.329
Ln TG Adjusted^1^ model	Q1 (DDS ≤ 4)	Ref		Ref		Ref	
	Q2 (DDS = 5)	0.019 (−0.023, 0.060)	0.384	0.051 (−0.012, 0.114)	0.112	−0.007 (−0.063, 0.048)	0.795
	Q3 (DDS = 6)	−0.016 (−0.063, 0.030)	0.487	0.003 (−0.063, 0.070)	0.922	−0.035 (−0.099, 0.030)	0.290
	Q4 (DDS ≥ 7)	−0.003 (−0.056, 0.050)	0.906	0.061 (−0.016, 0.138)	0.120	−0.070 (−0.143, 0.003)	0.061
TC Adjusted^1^ model	Q1 (DDS ≤ 4)	Ref		Ref		Ref	
	Q2 (DDS = 5)	0.009 (−0.059, 0.077)	0.794	0.053 (−0.038, 0.145)	0.252	−0.027 (−0.126, 0.072)	0.595
	Q3 (DDS = 6)	0.028 (−0.047, 0.102)	0.468	0.018 (−0.079, 0.114)	0.719	0.041 (−0.073, 0.155)	0.477
	Q4 (DDS ≥ 7)	0.002 (−0.083, 0.088)	0.954	0.057 (−0.054, 0.169)	0.316	−0.055 (−0.185, 0.075)	0.407
LDL Adjusted^1^ model	Q1 (DDS ≤ 4)	Ref		Ref		Ref	
	Q2 (DDS = 5)	−0.008 (−0.066, 0.049)	0.772	0.022 (−0.054, 0.098)	0.573	−0.033 (−0.118, 0.053)	0.456
	Q3 (DDS = 6)	0.006 (−0.058, 0.070)	0.854	−0.023 (−0.103, 0.056)	0.565	0.038 (−0.060, 0.137)	0.446
	Q4 (DDS ≥ 7)	0.010 (−0.063, 0.083)	0.782	0.045 (−0.047, 0.138)	0.340	−0.029 (−0.142, 0.084)	0.612
HDL Adjusted^1^ model	Q1 (DDS ≤ 4)	Ref		Ref		Ref	
	Q2 (DDS = 5)	−0.001 (−0.024, 0.022)	0.929	−0.003 (−0.037, 0.031)	0.855	−0.0002 (−0.032, 0.031)	0.990
	Q3 (DDS = 6)	**0.032 (0.006, 0.057)**	**0.014**	**0.045 (0.009, 0.080)**	**0.014**	0.019 (−0.017, 0.056)	0.299
	Q4 (DDS ≥ 7)	−0.020 (−0.049, 0.009)	0.182	−0.040 (−0.081, 0.001)	0.056	0.004 (−0.037, 0.045)	0.849
Ln HOMA-IR Adjusted^1,2^ model	Q1 (DDS ≤ 4)	Ref		Ref		Ref	
	Q2 (DDS = 5)	0.052 (−0.026, 0.130)	0.191	0.076 (−0.047, 0.198)	0.226	0.027 (−0.072, 0.127)	0.594
	Q3 (DDS = 6)	−0.034 (−0.121, 0.052)	0.435	−0.013 (−0.144, 0.117)	0.839	−0.049 (−0.163, 0.066)	0.406
	Q4 (DDS ≥ 7)	−0.013 (−0.113, 0.085)	0.788	−0.018 (−0.168, 0.132)	0.812	−0.003 (−0.134, 0.128)	0.965

Ref, reference category; Ln, natural-log transformed variable where the base of the log was 2.71828; WC, waist circumference; SBP, systolic blood pressure; TG, triglyceride; TC, total cholesterol; LDL, low-density lipoprotein; HDL, high-density lipoprotein; HOMA-IR, insulin resistance from Homeostasis Model Assessment; DDS, dietary diversity score; Q, quartile.

^1^Adjusted for gender, household wealth, and maternal education.

^2^Additionally adjusted for sample storage time, that showed a negative correlation with fasting insulin level (Spearman’s ρ = −0.2, *P* < 0.001). Statistically significant coefficients are presented in bold.

## Discussion

We explored cross-sectional associations of DPs derived from cluster analysis and DDS with conventional cardiometabolic risk indicators in a rural birth cohort. Four distinct clusters were identified: Traditional, Fish-dominant, Meat-dominant, and High-variety; and none was associated with the indicators overall. However, gender-stratified analyses revealed lower plasma TG levels among boys in the Fish- and Meat-dominant clusters than boys in the Traditional cluster. The associations of DDS quartiles with WC, SBP, and HDL were significant only among the boys as well. Compared to boys in the bottom quartile of DDS, higher WC, and SBP were observed among boys in the top quartile.

The diet clusters identified in our study aligned with those found among 9–15 years old adolescents (*n* = 30,702, mean age 11.6 years, 49.6% girls) in rural, northwest Bangladesh between 2015 and 2017 (45). The researchers identified five *a posteriori* DPs using latent class analysis of dietary data collected through a food frequency questionnaire in that study. Despite the methodological differences, the “traditional”, “moderately high meat”, and “most diverse” DPs identified in that study (45) resemble the Traditional, Meat-dominant, and High-variety DPs from the present study, respectively. High consumption of fish and low-carotenoid vegetables along with low consumption of animal-source foods including dairy characterized the DP considered traditional in both analyses. Moreover, a DP with higher diversity from consumption of nutrient-rich foods (e.g., vitamin-A-rich fruits and vegetables, animal-source foods and legumes) alongside unhealthy ultra-processed and deep-fried foods emerged in both analyses. Adolescents from relatively well-off households tended to have this DP in both studies. This potentially indicates a socio-economic gradient in rural Bangladesh (46) that allows consumption of a combination of nutrient-rich and empty-calorie foods among adolescents of relatively affluent families. Interestingly, the mean BAZ was about 0.1 SD higher among those with the diverse DP (“most diverse” and High-variety) than those with the traditional DP in both studies.

The *a posteriori* DPs did not show an overall association with the cardiometabolic risk indicators in this study. Several previous studies conducted among adolescents that employed cluster analysis (10, 13, 14, 47) also reported a general lack of association. This can be related to the variation in the relationship between diet and cardiometabolic risk across life phases. It has been shown that short-term associations between DPs and cardiometabolic risk indicators are attenuated among adolescents compared to adults in their fourth to sixth decades of life (48). Adolescents usually remain metabolically healthy around mid-adolescence, even including a proportion of those with obesity (49), and the impact of diet on specific cardiometabolic indicators may require a longer period to manifest (48). Moreover, the dietary share of foods that are major sources of saturated and trans fats, such as red and processed meat, full-fat dairy products and ready-to-eat/heat UPFs (50), were probably much lower in the rural setting of Matlab than in high-income settings. These foods contribute to more than 50% of the daily energy intake among adolescents in some high-income countries (51–53). Alternatively, DP-indicator associations might have been masked by differences in portion size and preparation style (e.g., deep versus shallow frying), and thus, in energy intake, as we did not quantify and adjust for total energy intake. Nevertheless, precise assessment of portion size is challenging in a rural setting where family members share food from a common bowl (54).

We found gender-specific negative associations of Fish- and Meat-dominant DPs with plasma TG. These translated into 8.5% lower TG among boys in the Fish-dominant cluster and 7.3% lower TG among boys in the Meat-dominant cluster than among boys in the Traditional cluster. The mechanism underlying this gender specificity remains unclear. While statistically significant, the “effect size” in terms of differences in TG level appeared small from a public health perspective (55). Adherence to a Mediterranean DP was inversely associated with serum TG among adolescent boys from Mexico City (19). Conversely, a “Rice and Kimchi” DP among South Korean adolescents with about 45% of the energy intake from white rice was associated with an elevated TG in both gender (14). The traditional DP in rural Bangladesh is dominated by white rice–a starchy staple with high glycemic index (GI) (56)–that contributes to up to 76% of the total energy intake among adolescents (57). Diets dominated by white rice have been linked to raised TG levels (58). It has been postulated that substantial intake of high-GI foods combined with relatively low intake of cholesterol and saturated fat, that is more likely in the Traditional cluster, may stimulate production of triglycerides (59). The negative associations with TG could be related to a replacement of white rice to some extent by protein intake from fish and meat in the Fish- and Meat-dominant clusters (60). Nonetheless, the qualitative recall precluded quantification of the dietary share of white rice across the clusters and further studies are needed to examine this assumption.

Some unanticipated associations emerged in relation to *a priori* DPs based on the quartiles of DDS. Having a DDS ≥ 7 was associated with higher SBP and the association was slightly amplified among the boys on gender stratification. A recent meta-analysis of studies among adults could not find any significant association between DDS and systolic or diastolic blood pressure (61). However, studies among adolescents involving *a priori* DPs generated conflicting results. Truthmann and colleagues (12) documented a positive association between dietary diversity and SBP among girls in a cross-sectional study (*n* = 5,198, mean age 15.1 years, 49.1% girls) from Germany. In contrast, DASH (Dietary Approaches to Stop Hypertension) scores showed no association with SBP in several studies (10, 62, 63). The instruments and the number and composition of food groups used to construct the *a priori* scores varied substantially between these studies, and limited the comparability. The positive associations with SBP and WC in our study may imply a greater intake of added salt and unhealthy fats from ultra-processed and deep-fried foods among the boys in the top DDS quartile. Among the boys in the top quartile (*n* = 151), the proportions of those who consumed these foods were high: 22.5% for ready-to-eat foods, 72.2% for confectioneries, 51.6% for savory snacks, 29.1% for SSB, and 56.9% for deep-fried foods (data not shown). Adjusting for total energy intake might have attenuated these associations. For instance, in a cross-sectional study among Iranian adolescents (*n* = 456, mean age 14 years, 58.5% girls), introduction of total energy intake in the regression model rendered the positive association between DDS and abdominal obesity (WC ≥ 85th percentile) statistically non-significant (17). The reason for the isolated association of having a DDS = 6 with higher HDL remains inconspicuous. The simple method used for calculating DDS in this study (33) satisfactorily predicted micronutrient adequacy among Bangladeshi adolescents (32). Nevertheless, it perhaps lacks the nuance and sensitivity required to capture associations between diet and cardiometabolic risk at mid-adolescence (61).

This study complements a growing body of literature (50) on the links between DPs and cardiometabolic risk among adolescents. Key strengths of the study include: a moderately large sample size based on a well-characterized, rural birth cohort (26), application of a double-pass method for dietary assessment that offered cost-effectiveness, high inter-rater reliability and low respondent burden while minimizing recall bias, and combination of *a priori* and *a posteriori* approaches to analyzing DPs. The findings are generalizable to adolescents in Matlab because of the area-wide recruitment of pregnant women in the MINIMat trial (25) and also to other rural, agrarian settings in Bangladesh due to the similarity in socio-cultural context. However, some critical limitations of this study need to be acknowledged. Drawing any causal inference from the associations would be erroneous owing to the cross-sectional design. We did not ascertain portion size and could not adjust the analyses for total energy intake. A single, 24-hour recall might not be entirely representative of the adolescents’ habitual dietary consumption. Under-reporting of sweet and savory snacks and SSBs in a 24-hour recall has been documented among LMIC adolescents (64, 65), but a recent validation study demonstrates the degree of under-reporting to be acceptable (64)–especially with larger samples. Although the decision to fix the number of clusters at four was based on the scree plot and the size and interpretability of clusters in Matlab context, it may limit comparability with other studies. Finally, we could not completely rule out residual confounding.

## Conclusion

We identified four DPs using cluster analysis in a birth cohort of adolescents in rural Bangladesh: Traditional, Fish-dominant, Meat-dominant, and High-variety clusters. No significant associations were observed between these clusters and selected cardiometabolic risk indicators, apart from small, negative associations of Fish- and Meat-dominant clusters with plasma TG among the boys. Furthermore, belonging to the top quartile of DDS was associated with higher SBP and WC among the boys. Associations between DPs and cardiometabolic risk indicators may require a time lag beyond mid-adolescence to be evident in a rural setting. Prospective studies with adjustment for energy intake are warranted to delineate the magnitude and direction of those associations.

## Data availability statement

The study under consideration availed data from the 15-year follow-up of the MINIMat (Maternal and Infant Nutrition Intervention in Matlab) trial. The 15-year follow-up was a large, collaborative project involving Uppsala University, Karolinska Institute, Finnish Institute for Health and Welfare, University of Oulu and International Centre for Diarrhoeal Disease Research, Bangladesh (icddr,b). Because of the statutory requirements, internal data policies and regulations existing in the collaborating bodies along with the over-arching General Data Protection Regulation (GDPR), the data must be stored in institutional repository (storage platforms) and cannot be made directly accessible without a review of the request for access to data. Data availability is further limited because the data contain information on gender and health-related and behavioral attributes, and thus, considered to be “sensitive personal data” as per GDPR. While the data are pseudonymized in accordance with GDPR, Supplementary material that can link the data to each study participant exist and are preserved following regulations in place at the collaborating bodies. Therefore, the data can be accessed only upon formal request that details the purpose of such request. Requests to access these datasets should be directed to the principal investigators of the MINIMat15y project: E-CE (email: lotta.ekstrom@kbh.uu.se) and AR (email: arahman@icddrb.org).

## Ethics statement

This study was a part of the 15-year follow-up of the MINIMat trial. The 15-year follow-up has been approved by the Ethical Review Committee at icddr, b in Dhaka, Bangladesh (PR-17029; date 2017/05/23). An additional approval has been obtained from the Ethics Review Authority (Etikprövningsmyndigheten) in Sweden (#2021–02796; date 2021/11/15). We obtained written informed consent from the mothers and assent from the adolescents.

## Author contributions

AR, MRI, and E-CE: conceptualization and design. MRI, E-CE, and KS: analyses. EK and MK: analysis of blood markers. AR, JP, SR, EK, MK, and E-CE: data curation. MRI: First complete draft. All authors contributed to the critical reviewing and redrafting, read, and approved the final manuscript.
