# Supplementary material for: Dietary patterns and indicators of cardiometabolic risk among rural adolescents: A cross-sectional study at 15-year follow-up of the MINIMat cohort
Source: Front Nutr. 2023 Jan 25;10:1058965. doi: 10.3389/fnut.2023.1058965 (PMC9905110; doi:10.3389/fnut.2023.1058965)
Supplement: Supplementary file 1 [file Data_Sheet_1.docx]

**Supplementary material**

**Supplementary Table 1.** Food grouping used for dietary assessment at 15-year follow-up of the MINIMat cohort. The cluster analysis incorporated all the food groups except Grains, white roots and tubers, and plantains. The first 10 groups were used to calculate dietary diversity score for each participant. Bengali names are italicized.

| **No.** | **Food group** | **Individual food items in the group** |
| --- | --- | --- |
| 1 | Grains, white roots and tubers, and plantains | Rice- cooked (*bhat*) and fried, puffed rice (*muri*), *panta*, wheat bread, paratha, chapatti, *luchi*, other items made from milled grains, maize, cassava, taro (*kochu mukhi*), green banana. |
| 2 | Vitamin A-rich vegetables, tubers and fruits* | Carrot, pumpkin, orange-fleshed sweet potato, mango (ripe), papaya (ripe), hog plum, watermelon. |
| 3 | Dark green leafy vegetables (DGLV) | Red amaranth, taro leaves, spinach, bottle guard leaves, mustard leaves, other locally available *shaak*. |
| 4 | Other (non-vitamin-A-rich) vegetables | Tomato, gourd, brinjal, *zhinga*, long bean, cucumber, teasle gourd, wax gourd, green papaya, cabbage, cauliflower, radish. |
| 5 | Other (non-vitamin-A-rich) fruits | Guava, banana, orange, apple, boroi, grapes, jackfruit, other fruits that are not vitamin A-rich. |
| 6 | Flesh and organ meat | Chicken, duck, beef, sheep, goat, pigeon, and liver, kidney or any other organ meat. |
| 7 | Egg | Chicken, duck, or quail eggs. |
| 8 | Fish | Rohu (*Rui*), *chitol*, *mrigal*, *shing*, *gojar*, *taki*, *puti*, tilapia, pangasius, hilsa, *kajuli*, *bashpata*, *koi*, *rani*, *bou*, dry fish, prawn etcetera. |
| 9 | Legumes, nuts and seeds | Beans, peas, lentils, hyacinth beans, pea seeds, groundnuts, peanuts. |
| 10 | Milk products | Milk, yogurt, *shemai*, *shuji*, *payesh*, *khir*, paneer, or other foods made with milk. |
| 11 | Ready-to-eat or “instant” foods (UPF) | Instant noodles (Maggi noodles), burgers, industrially mass-produced breads/loaves and buns. |
| 12 | Confectionery, sweets, and similar packaged products (UPF) | Biscuits or cookies, chocolates, candies; industrially mass-produced, packaged muffins and cakes, ice cream |
| 13 | Savory snacks (UPF) | Potato chips, crisps, chanachur, other salty/spicy packaged snacks, such as roasted peanuts, jhalmuri. |
| 14 | Sugar-sweetened beverages (UPF) | Soft drinks (Coca-Cola, Pepsi, Mirinda, Fanta), Jeera Pani (bottled, sweetened cumin water), energy drinks (Tiger, Shark, Speed, Power, etcetera), mix-and-drink sachet (such as Tang). |
| 15 | Deep-fried foods | *Shingara*, samosa, *puri*, *fuchka*, lentil fritters, french fries. |

*****Definition of Vitamin A-rich vegetables, tubers, and fruits was based on the FAO guideline (1). UPF: ultra-processed foods.

*1. Kennedy G, Ballard T, Dop M-C. Guidelines for measuring household and individual dietary diversity. Rome: FAO (2011).*

**Supplementary Figure 1: Scree plot**


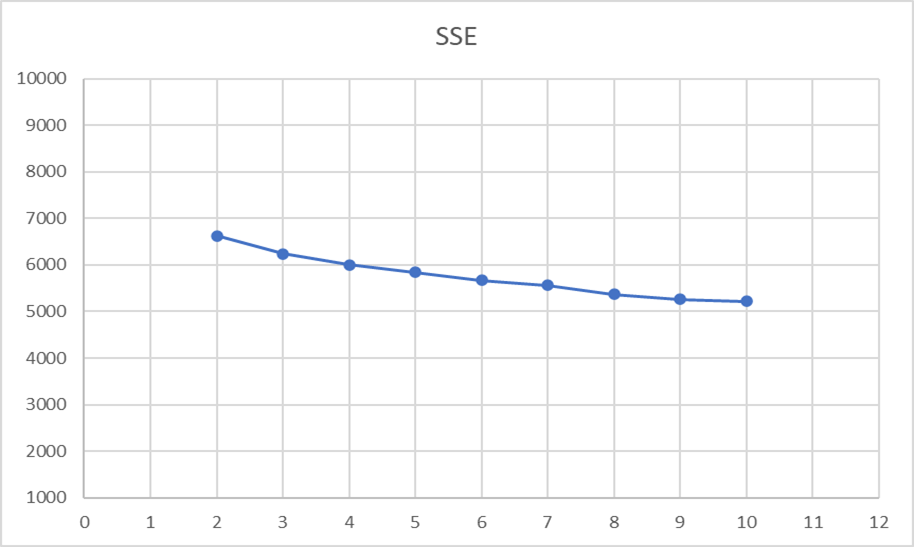


**Supplementary Figure 1.** A line graph showing the sum of squared errors plotted against number of clusters from simple *K*-means clustering based on dietary data (n = 2464). SSE: sum of squared errors.

**Supplementary Figure 2**

**Supplementary Figure 2**. Directed acyclic graph for the relationship of dietary patterns (DP) with indicators of cardiometabolic risk (CM indicators) prepared in dagitty.net. The pink arrows indicate biasing paths and the green arrows indicate causal paths. HH: household; Adol. edu: adolescents’ education; Mat. edu: maternal education.
